# Measurement of knowledge and attitudes concerning electroconvulsive therapy

**DOI:** 10.1192/bjb.2022.35

**Published:** 2022-08

**Authors:** Richard Braithwaite

**Affiliations:** Lead Consultant Psychiatrist for Neuromodulation, Sussex Partnership NHS Foundation Trust, Meadowfield Hospital, Worthing, UK. Email: richard.braithwaite@nhs.net

Cheung and colleagues' randomised controlled trial of strategies to improve attitudes toward electroconvulsive therapy (ECT)^1^ is very welcome, given the obstacles built upon ignorance that often prevent patients receiving what is an extremely safe and effective treatment.^2^

Interestingly, although there appeared to be some limited benefits from watching a video, the authors found that attitudes were no more positive and, more surprisingly, knowledge no better, in the group randomised to read a leaflet about ECT than in the control group given no information. They do not present any convincing explanation for this apparent lack of factual knowledge above baseline in a group of people who had just been educated on the very topic in question.

I suspect the answer lies in their choice of outcome measure. The questionnaire to test knowledge and attitudes used in their study of UK student participants is drawn from a similar trial conducted on arts students in the USA.^3^ In turn, that study had borrowed its questionnaire from an earlier Saudi trial which had used nursing students as subjects,^4^ but for the purposes of their study the US authors had removed from the questionnaire several items not covered by the leaflet of which they were testing the efficacy; they also extended the range of response options. It is notable that Cheung and colleagues had not similarly tailored the questionnaire to match the content of their own leaflet, instead using the US version.

However, most importantly, the questionnaire is simply unfit for purpose, as it is littered with factual inaccuracies, ambiguities and oddities, four of which are outlined below.

One item asks whether ‘ECT leads to permanent loss of memory,’ the supposedly correct answer being that is does not; in reality, objective evidence is inconclusive.^2^ One of the staunchest supporters of ECT, Harold Sackeim, has suggested it would be unreasonable to dismiss the experiences of patients who report such a phenomenon,^5^ many of whom anecdotally remain overwhelmingly positive about the treatment. It is unclear how the vague statement ‘ECT should be administered without obtaining patients' informed consent’ ought to be responded to; it was written in Saudi Arabia prior to the introduction of that country's first ever mental health legislation in 2014 and, according to the scoring schedule, it is a false statement, yet there is a cohort of seriously ill patients for whom it might be correct. Another item invites subjects to gauge their degree of agreement with the statement ‘I feel comfortable watching ECT being administered’. This item was aimed at the nursing students in the original Saudi trial but is wholly irrelevant for subjects attending a plethora of mainly non-healthcare courses at the University of Portsmouth. ‘ECT can be performed without anaesthesia’ is a correct statement on this questionnaire, but presumably the information given to subjects in the intervention groups would have made clear that this is not acceptable practice in the UK, setting them up to give the ‘wrong’ answer. There are several other examples of poorly worded items on the questionnaire.

Despite being relatively well informed on ECT and a strong proponent of the treatment, I score well below full marks on both the knowledge and attitude scales of this questionnaire, owing to its poor construction. A psychiatrist was consulted by the authors at the design stage of this study, but notably he is employed by an organisation that does not provide ECT. It is perhaps regrettable that no expert in the field was asked to provide advice to the researchers, especially with respect to the choice of outcome measure. Because of this shortcoming, I fear it is impossible to draw any useful conclusions from this study.

## References

[1] Cheung O, Baker M, Tabraham P. Improving attitudes toward electroconvulsive therapy. BJPsych Bull 2022; 46(1): 4–10. 33583475 10.1192/bjb.2021.5 PMC8914918

[2] Kirov G, Jauhar S, Sienaert P, Kellner CH, McLoughlin DM. Electroconvulsive therapy for depression: 80 years of progress. Br J Psychiatry 2021; 219(5): 594–7. 35048827 10.1192/bjp.2021.37

[3] Hoffman GA, McLellan J, Hoogendoorn V, Beck AW. Electroconvulsive therapy: the impact of a brief educational intervention on public knowledge and attitudes. Int Q Community Health Educ 2018; 38(2): 129–36. 29277138 10.1177/0272684X17749939

[4] Dawood E, Selim A, Khalil A. Electroconvulsive therapy: effect of an educational experience on nursing students’ knowledge and attitudes. J Nurs Educ Pract 2013; 3(9): 123–9.

[5] Sackeim HA. Autobiographical memory and electroconvulsive therapy: do not throw out the baby. J ECT 2014; 30(3): 177–86. 24755727 10.1097/YCT.0000000000000117 PMC4141894

